# Accelerated corneal endothelial cell loss in two patients with granulomatosis with polyangiitis following phacoemulsification

**DOI:** 10.1186/s12886-020-01752-y

**Published:** 2020-12-07

**Authors:** Fang-Chi Hsiao, Hung-Ta Chen, Kuan-Jen Chen, Yi-Jen Hsueh, Yaa-Jyuhn James Meir, Tsai-Te Lu, Chao-Min Cheng, Wei-Chi Wu, Hung-Chi Chen

**Affiliations:** 1 Department of Ophthalmology, Chang Gung Memorial Hospital, No. 5, Fuxing Street, Guishan District, Linkou, Taoyuan, 33305 Taiwan; 2 Department of Medicine, Chang Gung University College of Medicine, Taoyuan, Taiwan; 3 Department of Internal Medicine, Taipei City Hospital- Heping Branch, Taipei, Taiwan; 4 Center for Tissue Engineering, Chang Gung Memorial Hospital, Linkou, Taiwan; 5 Department of Biomedical Sciences, Chang Gung University College of Medicine, Taiyuan, Taiwan; 6 Institute of Biomedical Engineering, National Tsing Hua University, Hsinchu, Taiwan

**Keywords:** Granulomatosis with polyangiitis, Phacoemulsification, Cataract, Corneal, Endothelial cell density (ECD)

## Abstract

**Background:**

Generally, the loss rate of human endothelial cells (HCEC) in routine cataract surgery is 8.5%. When the corneal endothelial cells density (ECD) drops, the HCEC may decompensate to keep cornea dehydration which leads to corneal edema. Granulomatosis with polyangiitis (GPA) is an uncommon autoimmune disease involving multiple organs including eyes such as conjunctivitis, scleritis, uveitis, and corneal ulcer. In this study, we report two cases of GPA whose corneal ECD decreased significantly after phacoemulsification cataract surgery.

**Case presentation:**

In the first case of 69-year-old male with GPA, the ECD dropped 39.6% (OD) four months after phacoemulsification and 38.1% (OS) six months postoperatively respectively. At the final follow-up, the residual ECD was only 55% in the right eye in the 49^th^ month, and 56% remained in the left eye in the 39^th^ month. In the second case of 54-year old female, left ECD dropped 63.9% at the 4^th^ month after surgery and 69.6% ECD remained at the 15^th^ month postoperatively while similar ECD of right eye before and after left eye surgery.

**Conclusion:**

Extensive preoperative ophthalmic evaluation and meticulous postoperative inflammation control should be applied to prevent severe loss of HCEC in GPA patients.

## Background

Granulomatosis with polyangiitis (GPA) is a systematic autoimmune disease with prevalence of 0.003% in America [1, 2], with 50–60% patients presenting with ocular manifestation lifetime [3]. The suspected etiology is activated dendritic cells that present autoantigen and prime type 1 T helper cells inducing macrophage migration, maturation and causing granuloma and tissue destruction. Chronic T cells activation promotes B cells secretion of antineutrophil cytoplasmic antibodies (ANCAs) targeting proteinase 3 (PR3) or myeloperoxidase (MPO) which trigger neutrophil activation and lead to vasculitis [4]. The clinical manifestations include flu-like symptoms, hearing loss, otitis media, bloody rhinorrhea, sinusitis, pulmonary disease, renal, cutaneous, neurologic problems and also conjunctivitis, scleritis, corneal ulceration, or uveitis [5, 6]. Importantly, most patients with GPA and ocular comorbidity are visually or systemically impaired [3].

Physiologically, the corneal endothelium consists of a monolayer of human corneal endothelial cells (HCEC), which remain contact-inhibited or non-proliferative, and endothelial cell density (ECD) diminishes gradually since birth until death [7]. The average ECD is around 2500 cells/mm^2^ in older adults [8]. When the ECD falls below 400–700 cells/mm^2^, the HCEC fail to pump the water out the cornea which ends up with corneal edema [9]. Pathologically, the loss rate of HCEC in routine cataract surgery is 8.5% [10], while that in patients with low ECD ranges from 5.1% to 12.1% [11, 12]. By contrast in the diabetic population, phacoemulsification-induced HCEC loss ranges from 6.2% up to 29.7% [13].

Here, we report a 69-year-old man with GPA suffering from significantly declined ECD up to 45% after phacoemulsification in the following-up of 49 months. Additionally, we report a 54-year-old woman with GPA suffering from declined ECD to 36.1% four months after cataract surgery, whose ECD recovered to 69.6% of the original level with ascorbate drops for 11 months.

## Case presentation

### Case 1

A 69-year-old Taiwanese Han male with GPA presented to our ophthalmologic clinic with redness, foreign body sensation, and pain in the right eye for two months. Before this episode, multiple sinusectomy was done for chronic aspergillus rhinosinusitis three months ago.

**Fig. 1:**
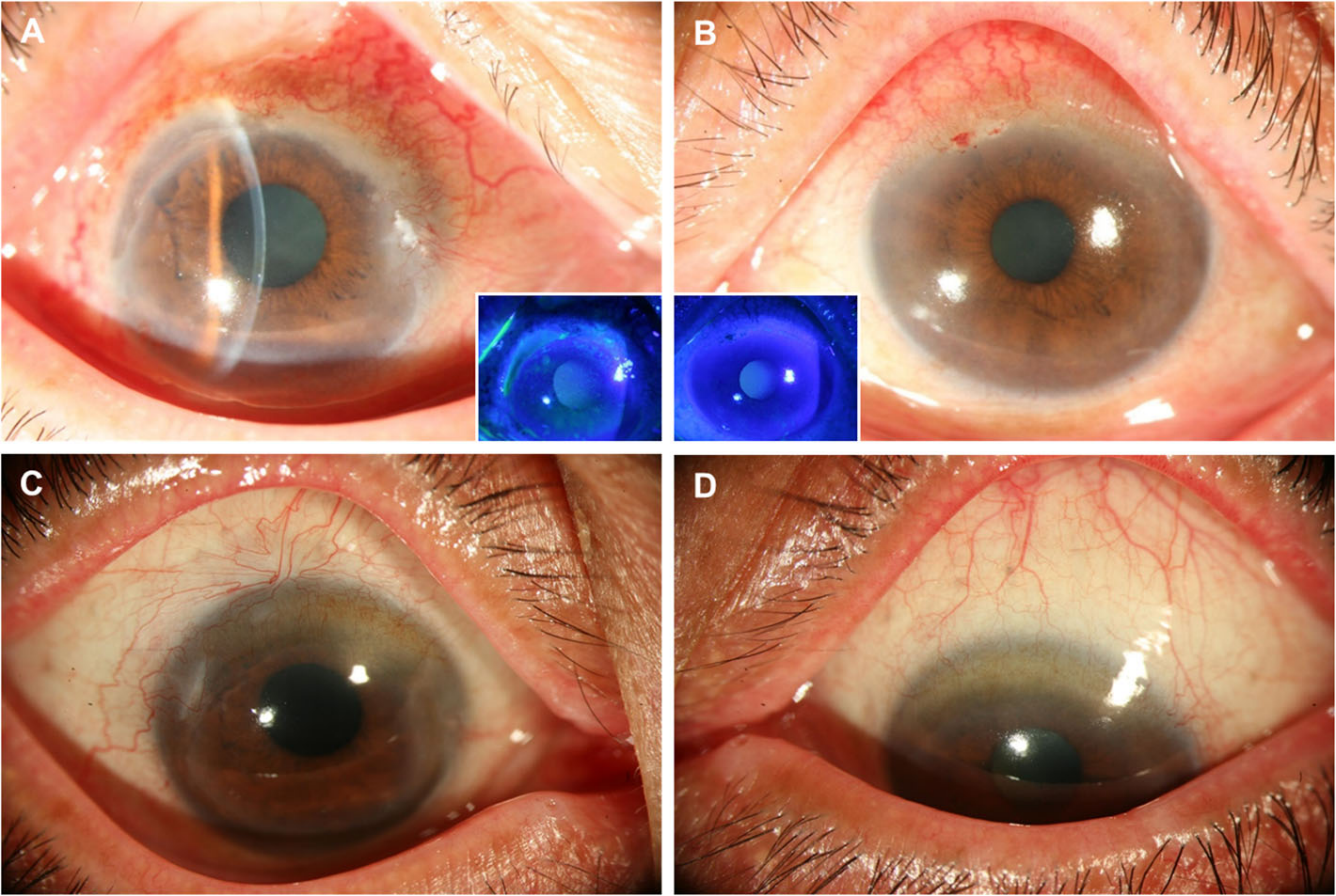
Serial external eye photography of Patient 1 before and after diagnosis of granulomatosis with polyangiitis (GPA). **a**. Initial presentation of superior nasal conjunctiva congestion (OD) with a 5-mm superior conjunctival ulceration and a 9 to 12 o’clock peripheral ulcerative keratitis was noted with visual acuity (VA) was 0.09. The insert shows fluorescein staining of some tiny epithelial defects and diffuse superficial punctate staining of the cornea. **b**. Initial presentation of superior conjunctival congestion and limbo-corneal neovascularization (OS) was noted with VA of 0.1. The insert shows fluorescein staining of mild superficial punctate staining of the cornea. **c.d**. Six months after initial presentation (i.e. 4 months after right eye cataract surgery), resolution in conjunctiva congestion (OU), peripheral ulcerative keratopathy (OD) and limbo-corneal neovascularization (OS) was noted with VA of 0.6 (OD) and 0.2 (OS) respectively

Ophthalmologic examination revealed visual acuity of 20/250 (OD) and 20/100 (OS), with bilateral congestion and an arcuate corneal ulceration between the 9 and 12 o’clock positions, as well as a 5-mm superior conjunctival ulceration in the right eye (Fig. 1a and b). Prednisolone (1%) eye drops and lubricant were prescribed to ameliorate impending perforation of the corneal ulcer, resulting in reduced reaction in the anterior chamber. Oral methylprednisolone 16 mg/day was also given initially to decrease the disease activity to facilitate subsequent conjunctival biopsy, which only showed necrosis and acute inflammation, but failed to show multinucleated giant cells, granuloma, or microangiopathy.

Hydroxychloroquine 400 mg was also added for 2 months under the suggestion of a rheumatologist, which was later shifted to prednisone 10 mg/day. One month later, visual acuity recovered to 20/60 (OU), and the PUK resolved. Free from further ocular symptoms and signs for one-year under the control of prednisolone 10 mg/day and methotrexate 7.5 mg/week, visual acuity decreased to finger counting (OD), which was caused by cataract after screening. In addition to grade 3 nuclear sclerosis and cortical opacity, dense posterior subcapsular cataract was also noticed probably due to long-term corticosteroid use. To prevent possible autoimmune-related corneal complications from intraocular surgery, we kept topical prednisolone acetate 1% and artificial tears in addition to planned medical treatment. Blood tests for immune activity were also relatively low before cataract surgery. No perioperative complication, increasing intraocular pressure nor postoperative inflammation were noted. The anterior chamber was deep and clear after surgery. There was mild arcuate neovascularization around the phacoemulsification wound and gradually subsided (Fig. 1c and d).

His visual acuity improved to 20/30 (OD). Ten months after right eye cataract surgery, his left eye visual acuity became 0.01 due to nuclear sclerosis and cortical opacification. The VA (OS) improved to 20/25 after cataract surgery. As right cataract surgery, no perioperative complication, increasing IOP nor postoperative inflammation were noted. The anterior chamber was deep and clear while slight arcuate neovascularization around the phacoemulsification wound was noted for few weeks.

**Fig. 2:**
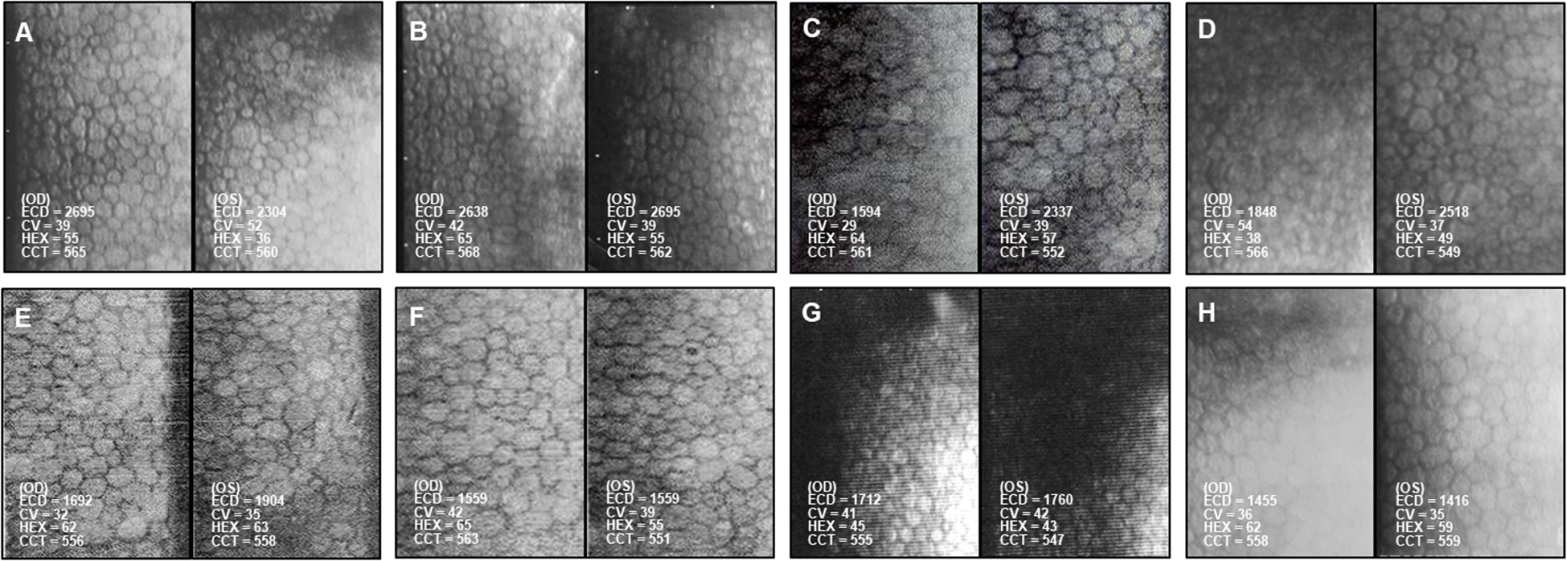
Serial imaging of the corneal endothelium before and after cataract surgery in the right eye (**a-d**) and after cataract surgery in the left eye (**e–h**) of Patient 1. Specular microscopy showed changes in endothelial cell density (ECD), coefficient of variation (CV), percentage of hexagonal cells (HEX), and central corneal thickness (CCT) at the following time points. **a**. Fourteen months prior to cataract surgery (OD). **b**. Two weeks prior to cataract surgery (OD). **c**. Four months after cataract surgery (OD). **d**. Seven months after cataract surgery (OD). **e**. Six weeks after cataract surgery (OS), i.e. 12 months after cataract surgery (OD). **f**. 6 months after cataract surgery (OS), i.e. Seventeen months after cataract surgery (OD). **g**. Fourteen months after cataract surgery (OS), i.e. 25 months after cataract surgery (OD) **h**. Thirty-nine months after cataract surgery (OS), i.e. 49 months after cataract surgery (OD)

The cataract surgeries (OU) were performed by an experienced surgeon (Chen HC) using standard techniques [14, 15]. Total ultrasound (US) power and time was 28.1 mJ and 94 s for the right eye, and 24.5 mJ and 76 s for the left eye using continuous and torsional energy power setting named Ozil with Alcon Laboratory #9056 Micro Tip Kelman 45 Degree phaco tip. The wound size was 2.65 mm. Preoperatively, ECD (OD = 2638 cells/mm^2^, OS = 2695 cells/mm^2^) was within normal range (Fig. 2a and b). After the right cataract surgery, the ECD dropped 39.6% in the 4^th^ month (Fig. 2c) and recovered to only 29.9% cell loss in the 7^th^ month (Fig. 2d), while the left ECD was still around its baseline. After the left cataract surgery, we found the ECD decreased to similar level of his right eye (Fig. 2e), approximately 44% loss binocularly in the 39 months following up (Fig. 2h).

### Case 2

A 54-year-old Taiwanese Han female with hypertension under medications control for 20 years and GPA with steroid and Imuran control for 3 years presented progressively blurred vision (OS) for several years.

Ophthalmologic examination revealed visual acuity of 20/28.5 (OD) and 20/200 (OS) without remarkable abnormalities in the anterior and posterior segments, except for grade 1 nuclear cataract assessed by the Lens Opacities Classification System III (LOCS III). Ten months later, her nuclear cataract became grade 2 and phacoemulsification and intraocular lens (OS) was performed in another 6 months later. The patient’s anterior chamber was shallow and clear before and after surgery. No obvious postoperative inflammation nor increasing IOP was noted compared with her baseline condition.

**Fig. 3:**
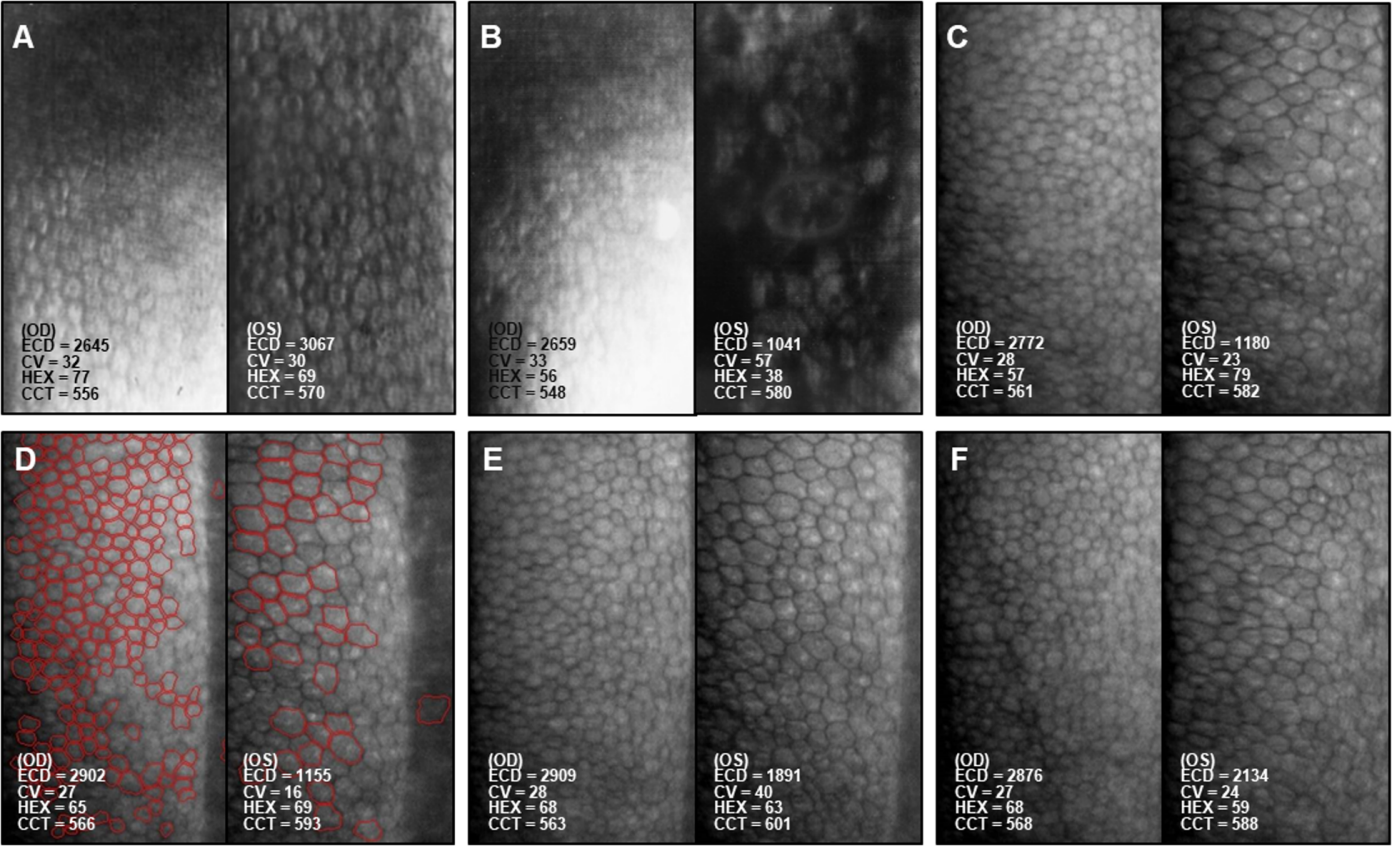
Serial imaging of the corneal endothelium before and after cataract surgery in the left eye (**a-f**) of Patient 2. Specular microscopy showed changes in endothelial cell density (ECD), coefficient of variation (CV), percentage of hexagonal cells (HEX), and central corneal thickness (CCT) at the following time points.**a**. One month prior to cataract surgery (OS). **b**. One month after cataract surgery (OS). **c**. Four months after cataract surgery (OS). **d**. Seven months after cataract surgery (OS), i.e. 3 months after topical use of ascorbic acid (OS). **e**. Nine months after cataract surgery (OS), i.e. 5 months after topical use of ascorbic acid (OS). **f**. Fifteen months after cataract surgery (OS), i.e.11 months after topical use of ascorbic acid (OS)

The cataract surgery was performed by an experienced surgeon (Chen HC). Total US power and time was 15.05 mJ and 50.2 s with the same facility and model in case 1. Tobramycin 0.3% + dexamethasone 0.1% suspension and oral mefenamic acid 1 g were prescribed for prophylactic use and pain control. Her ECD was 2645 cells/mm^2^ (OD) and 3067 cells/mm^2^ (OS) 1 month before left cataract surgery which was in normal range (Fig. 3a). Her ECD became 2659 cells/mm^2^ (OD) and 1041 cells/mm^2^ (OS) which was only 34.0% remained at the 1^st^ month after surgery (OS) (Fig. 3b) and following ECD were 2772, 2902, 2909, 2876 cells/mm^2^ (OD) and 1108, 1155, 1891, 2134 cells/mm^2^ (OS) at the 4^th^, 7^th^, 9^th^ and 15^th^ month (Fig. 3d-f).

We tried to find possible strategy to rescue the loss HCEC and studies showed ascorbic acid might help [16]. Topical ascorbic acid (10 mg/mL, qid) was administered since 4^th^ month after surgery for her left eye. At the last follow-up at the 15^th^ month, ECD totally lost 30.4% with visual acuity of 20/25(OD) and 20/100 (OS) and unremarkable ocular examination results which rescued 32.0% of ECD loss than that in the 4^th^ month.

## Discussion and conclusions

Previously known as Wegener's granulomatosis, GPA is an ANCA-associated autoimmune disease involving multi-organ damage and is fatal without treatment. In this report, the patients had most classic manifestations of GPA and gradually developed to hypertension, and even cataract due to long-term use of steroid. Besides, PUK was also presented concurrently in one patient [17].

For a patient with GPA, long-term use of corticosteroid may accelerate the formation of posterior subcapsular cataract, with several theories [18]. Thorough systemic review, close follow-up, and control of local inflammation are mandatory before intraocular surgery because autoimmune-related corneal melting can occur spontaneously or after cataract extraction [19, 20]. There is also research aiming to discover specific antibodies in patients with autoimmune diseases that are responsible for corneal melting [21]. When the auto antibodies bind to specific corneal antigen forming immune complex, the classical pathway of complement was activated. Inflammatory cells are driven to peripheral cornea and produce cytokines inducing stromal keratocytes to release matrix metalloproteinases which subsequently dissolve the cornea [22–24].

In this case, we observed an abnormal pattern of corneal endothelial cell loss after cataract surgery, even though that one patient had concomitant diabetes under stable metformin therapy and a long-term serum HbA1c level of 6.0 to 6.5%. In addition, his inflammatory status was relatively stable based on preoperative examinations of serum C reactive protein (CRP) quantity and erythrocyte sedimentation rate (ESR) which were controlled around 10 mg/L and 25 mm/hour one month after the right cataract surgery.

Though this is only a case report, the patient’s binocular ECD decreased approximately 40% four months postoperatively and ended up around 44% in the forty months follow-up and another patient’s ECD decline 38.3% nine months after surgery which was apparently higher than that of 1.4% to 18.4% in non-diabetic and 6.2% to 29.7% in diabetic patients three months postoperatively shown in a meta-analysis study [13]. We speculate that the patient’s corneal endothelial cells were more susceptible to intraocular surgery than those in the normal population and even the diabetic patients owing to underlining GPA. In other word, the marked ECD decline was less likely related to autoimmune reaction aroused by cataract surgery per se since there was no severe postoperative local inflammation and no significant ECD decline in the left eye after the right cataract surgery. Also, no right eye ECD decreased in the second patient after left cataract surgery for 15 months. In the second case, tropical ascorbate sodium was given since the 4^th^ month after phacoemulsification which showed gradually increasing ECD to the last follow up at the 15^th^ month. Perioperative topical ascorbic acid (AA) has been shown preventive effects of corneal endothelial dysfunction in high-risk patients undergoing phacoemulsification [16]. AA has shown promoting proliferation effects in other human cells [25, 26]. *In vitro* evidence also shed light on the proliferative effects of AA on corneal endothelial cells [27]. Besides, there is clinical evidence of protective effect of AA on HCEC recently [16].

We have illustrated two rare cases of an old-aged male patient with GPA and PUK and a middle-aged female with GPA undergoing uneventful phacoemulsification but with marked loss of corneal endothelial cells. In the long-term follow-up, ECD decline was even more the that in the general population and diabetic group [13]. Collectively, we advocate extensive preoperative ophthalmic evaluation and meticulous postoperative inflammatory control to prevent severe corneal endothelial cell loss in such patients.

## Data Availability

All data generated during this case report are included in this published article.

## Abbreviations

AA: Ascorbic acid
ANCAs: Antineutrophil cytoplasmic antibodies
CRP: C reactive protein
ECD: Endothelial cells density
ESR: Erythrocyte sedimentation rate
GPA: Granulomatosis with polyangiitis
HCEC: Human corneal endothelial cells
MPO: Myeloperoxidase
PR3: Proteinase 3
